# The gastric isthmus from D+ and D− broiler lines divergently selected for digestion efficiency shows histological and morphological differences^1^

**DOI:** 10.3382/ps.2013-03756

**Published:** 2014-04-28

**Authors:** N. Rideau, E. Godet, C. Combémorel, M. Chaudeau, B. Carré, S. Mignon-Grasteau

**Affiliations:** UR083 Recherches Avicoles, INRA, F-37380, Nouzilly, France

**Keywords:** chicken, digestibility, selection, gastric isthmus, histology

## Abstract

Previous results suggested that the gastric function plays a paramount role in digestive efficiency differences between D+ and D− broiler lines divergently selected for AME_n_ (more feed efficient and less feed efficient, respectively). In this paper we show an histological analysis of the gastric isthmus located between the proventriculus and the gizzard in the D+ and D− lines. Cross-sections were performed using a cryostat (Leica CM30505) and stained with a routine procedure using Mayer’s Hematoxylin and Eosin Stain. The surface and shape of the constitutive gastric isthmus tissues were quantified using the image analysis software Image J. The lumen occupied 11% of the whole D− isthmus cross-sectional area against 24% for D+ (*P* < 0.01). The mucosa relative area (cm^2^/total cross-sectional area) was higher in D− than in D+ [47% (D−) and 39% (D+), *P* < 0.01]. It was significantly more oval and more folded on the lumen side in D− than in D+ chickens; the muscle layer (muscularis mucosae) of the mucosa was relatively more developed in D− than in D+ (16 and 11% of the section, respectively; *P* < 0.01). A relationship between these observations and increased gastric motility reported in D− compared with D+ is discussed.

## INTRODUCTION

The capacity of broilers to digest a wheat diet was shown to be heritable (h^2^ = 0.37; Mignon-Grasteau et al., 2004). Two divergent lines (**D+** and **D−**; more feed efficient and less feed efficient, respectively) were consequently selected on the basis of the AME_n_ value of a wheat diet measured at 3 wk of age. After 8 generations of selection, the differences between lines reached 32, 29, 42, and 10% for diet AME_n_ value and digestibility of starch, lipids, and proteins, respectively (Rougière and Carré, 2010).

Differences in digestive efficiency between the 2 lines were associated with gastrointestinal anatomical and physiological modifications: the gizzard was heavier and the intestine lighter in the D+ line compared with the D− line (Garcia et al., 2007; Rougière et al., 2009; de Verdal et al., 2010, 2011). The average retention time in the gizzard was 2 times higher in the D+ line than in the D− line, and the digestive transit was accelerated in D− chickens compared with D+ (Rougière and Carré, 2010). Gastric motility as measured with a strain gauge transducer also differed with a higher responsiveness of the gizzard to environment variations in D+ than in D− birds and a failure in the gizzard relaxation process during rest in D− birds (Rougière et al., 2012). Intestinal adaptation to compensate for the low functionality of the gastric area in D− birds was suggested by increased small intestinal weight (Garcia et al., 2007; Rougière et al., 2009) and modifications of intestinal epithelial area and muscle layer thickness assessed by histological analyses (de Verdal et al., 2010).

The avian stomach consists of 2 distinct structures, the proventriculus or glandular stomach and the ventriculus (gizzard) or muscular stomach. The gastric isthmus is a short constricted region separating the glandular and muscular portions of the stomach. Several studies from Duke’s laboratory reported that the entire myenteric plexus associated with the gastric isthmus must remain intact for proper gastroduodenal motility to take place in domestic turkeys (Chaplin and Duke, 1988; Hall and Duke, 2000). These results confirmed the existence of a neurogenic pacemaker driving the gastroduodenal cycle within the myenteric plexus associated with the isthmus.

Interstitial cells of Cajal are specialized cells in smooth muscle organs that generate and propagate pacemaker activity (spontaneous electrical rhythms of the gut known as slow waves), and receive inputs from motor neurons and excitatory cholinergic and inhibitory neural inputs from the enteric nervous system associated with mechanosensors through stretch receptors that modulate membrane potential and electrical slow wave frequency (Sanders and Ward, 2007). It was further discovered that interstitial cells of Cajal contain the product of the c-kit oncogene (Huizinga et al., 1995). Lecoin et al. (1996) identified c-kit positive cells in the chicken embryo with c-DNA probes and the disruption of peristaltic activities with antibodies to the c-kit product. The occurrence of c-kit positive cells was abundant at the junction between the proventriculus and gizzard and at the cranial and caudal poles of the gizzard, corresponding exactly to the location of the neurogenic pacemaker in the isthmus region of the turkey.

Looking for qualitative and quantitative gastric physiological differences between the 2 lines, we characterized the gastric isthmus of D+ and D− lines through histological cross-sections as a preliminary approach to get further insight into gastric innervation and regulatory role of the gastric isthmus possibly implicated in differences between D+ and D− lines.

## MATERIALS AND METHODS

### Birds

Seventy-two birds (36 males and 36 females) from the D+ and D− lines resulting from 8 selection generations (Mignon-Grasteau et al., 2004) were individually weighed at hatching and placed in groups on wood shavings from 0 to 11 d; the bird density was 12 chickens per m^2^. After 13 d, chicks were randomly allocated to individual cages to monitor individual food intake. The environmental conditions were controlled for ventilation, lightning program (24L:0D from 1 to 7 d and 18L:6D from 8 to 23 d, dark periods beginning at 23 h) and temperature (from 33°C at 1 d gradually decreasing to 21°C at 22 d). The birds had free access to water and food. The chickens were fed a high-viscosity wheat-based diet (20.5% proteins and 2,943 kcal∙kg^−1^, consisting of 52.5% of Rialto wheat, as used in the selection experiment (Mignon-Grasteau et al., 2004), given ad libitum as pellets. All birds were individually weighed at hatching, at 12 and 22 d. Total individual food intake was recorded from 12 to 22 d, and feed conversion ratio (**FCR**) was calculated.

### Gastric Isthmus Sampling

At the end of the experiment (23 d), 60 birds (15 males and 15 females from each line) were weighed and killed by CO_2_ inhalation. The gastric compartment was excised immediately after birds were killed, and it was cleaned and emptied. The gastric isthmus was dissected, washed with a physiological solution (PBS), weighed, frozen in isopentane, and stored at −80°C until analysis. The proventriculus and the gizzard were emptied, washed, and preserved at 4°C during 4 h to be defatted and weighed.

All procedures were approved by the French Agricultural Agency and the Scientific Research Agency and conducted in accordance with the guidelines for Care and Use of Agricultural Animals in Agricultural Research and Teaching.

### Histological Sampling and Analysis

**Figure 1.**
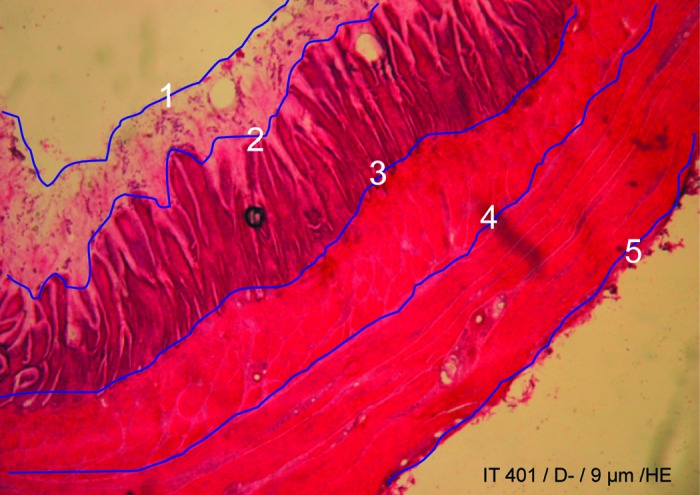
The different layers of a gastric isthmus cross-section [D− (less feed efficient) chicken; hematoxylin-eosin, HE]. The delineated profile of the layers for Image J (http://rsbweb.nih.gov/ij/) quantification is indicated with black lines (blue in color version). 1: Lumen, 2: mucous, 3: epithelium, 4: muscularis mucosae, 5: muscularis externa. Color version available in the online PDF.

The histological study was carried out on 10 randomly selected gastric isthmus samples from each line. The isthmus was cut (from the lower part of the proventriculus to the upper part of the gizzard) at −20°C into 9-µm-thick cross-sections using a cryostat (CM30505, Leica Microsystems, Wetzlar, Germany), and sections were placed on glass slides. A routine staining procedure was carried out using Mayer’s Hematoxylin and Eosin Stain (Sigma-Aldrich, Saint-Quentin Fallavier, France). The preparations were then mounted between slides and coverslips with the addition of an aqueous agent for microscopy (Eukitt, CML Distributor, Neumours, France). The slides were examined using a binocular magnifying glass (SMZ-U, Nikon, Tokyo, Japan) fitted with a camera (D70s, Nikon). Images were captured for each sample with a final ×15 magnification. A representative gastric isthmus cross-section corresponding to the narrowest area composed of smooth muscles deprived of proventricular gland was selected and quantified using the Image J (http://rsbweb.nih.gov/ij/) image analysis software. The different tissue layers were manually delimited (Figure 1), and the area of the layers, shape (circularity index), and folding (solidity index) of contours were calculated. Circularity equals 1 for a circular contour and becomes progressively smaller (toward 0.1) as the structure flattens. Solidity equals 1 when no folding occurs and tends toward 0.1 as folding increases similarly; a solidity of 1.0 was assigned when there was no folding of the tissue and approached 0.1 as folding increased.

### Statistical Analyses

Statistical analyses were performed using Mann and Whitney’s test for nonpaired values (Statview software program, version 5; Statview, Abacus Concepts Inc., Berkeley, CA). Results are expressed as the mean ± SEM. A *P*-value of less than 0.05 was considered significant.

## RESULTS

### Proventriculus and Gizzard Morphology

Characteristics of the chickens used for the study are presented on Table 1. At hatch there was no difference in the chicken BW (*P* > 0.05). At 22 d, chickens of the D− line were 15% lighter (*P* < 0.001) than those of the D+ line but consumed 20% more food (*P* < 0.001) than D+ chickens, leading to a 47% higher (*P* < 0.001) FCR in D− line. The relative weight of the proventriculus and the gizzards were respectively 14 and 23% (*P* < 0.001) lighter in D− birds than in D+ birds. These data were in agreement with previous results obtained on these lines (Garcia et al., 2007; de Verdal et al., 2010).

**Table 1.** Means (±SEM) of BW (kg), food conversion ratio (FCR), and relative weight (g∙kg^−1^) of the proventriculus and gizzard in 22-d-old chicks from both divergent lines^1^

Item	n	D+	D−	*P*-value
BW (hatch)	10	0.0357 ± 0.0009	0.0364 ± 0.0008	NS
BW (22 d)	10	0.476 ± 0.007	0.405 ± 0.012	***
FCR (12−22 d)	10	1.58 ± 0.06	2.33 ± 0.18	***
Proventriculus weight	10	11.0 **±** 1.0	9.5 **±** −0.5	***
Gizzard weight	10	31.3 **±** 1.3	24.2 **±** −0.9	***

^1^D+ and D−: more feed efficient and less feed efficient, respectively.

NS > 0.05; ***P < 0.001.

### Gastric Isthmus Histology

**Figure 2.**
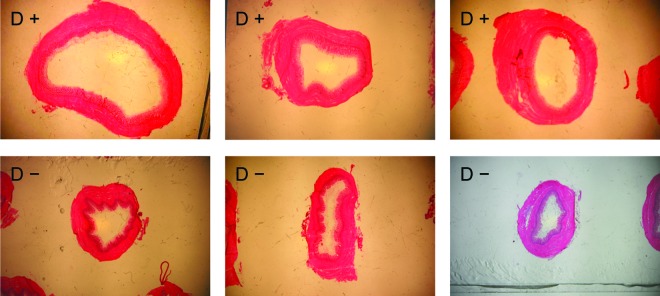
Typical cross-section macroscopic view of the gastric isthmus from 3 D+ and 3 D− (more feed efficient and less feed efficient, respectively) chickens. Photographs were captured using a binocular magnifying glass (SMZ-U, Nikon, Tokyo, Japan; hematoxylin-eosin, X15). Color version available in the online PDF.

The major areas of the gastric isthmus (Figure 1) included the lumen, the mucosa (with mucous, epithelium, lamina propria, and muscularis mucosa layers), the submucosa (connective tissue), the muscularis externa, and the serosa (connective tissue). The muscularis externa was composed of circular smooth fibers only; the absence of longitudinal smooth fibers at this level was in agreement with Bennett and Cobb (1969), showing no longitudinal muscle in the gizzard. There was no proventricular gland in the gastric isthmus area. Gastric isthmus cross-sections were wider in D+ than in D− chickens; the lumen also appeared wider and the musosal epithelium presented fewer folds or no folds in D+ than in D− chickens (Figure 2). These differences have been quantified using Image J as presented on Tables 2 and 3.

**Table 2.** Means (±SEM) of absolute (cm^2^) and relative area (area of the layer to the total cross-section area) of the different layers of the gastric isthmus from the D+ and D− chickens (more feed efficient and less feed efficient, respectively)^1^

	Area (cm^2^)				Relative area (cm^2^∙cm^−2^)		
Item	D+	D−	*P*-value		D+	D−	*P*-value
Cross-section	0.93 ± 0.08	0.56 ± 0.06	**				
Lumen	0.24 ± 0.05	0.06 ± 0.01	***		0.24 ± 0.03	0.11 ± 0.02	**
Mucous	0.08 ± 0.01	0.06 ± 0.01	NS		0.09 ± 0.01	0.09 ± 0.02	NS
Epithelium	0.18 ± 0.01	0.12 ± 0.01	**		0.22 ± 0.01	0.19 ± 0.01	NS
Muscularis mucosae	0.10 ± 0.01	0.09 ± 0.01	NS		0.11 ± 0.01	0.16 ± 0.01	**
Mucosae	0.36 ± 0.02	0.26 ± 0.03	*		0.39 ± 0.02	0.47 ± 0.02	**
Muscularis externa	0.33 ± 0.02	0.24 ± 0.03	**		0.37 ± 0.02	0.42 ± 0.02	NS

^1^The area was calculated using the Image J (http://rsbweb.nih.gov/ij/) image analysis software.

NS > 0.05; *P < 0.05; **P < 0.01; ***P < 0.001.

**Table 3.** Means (±SEM) of the circularity and solidity index of the contours of the different layers of the gastric isthmus from the D+ and D− chickens (more feed efficient and less feed efficient, respectively)^1^

	Circularity				Solidity		
Item	D+	D−	*P*-value		D+	D−	*P*-value
Lumen	0.67 ± 0.04	0.41 ± 0.08	*		0.90 ± 0.01	0.65 ± 0.08	**
Mucus	0.73 ± 0.04	0.40 ± 0.05	***		0.92 ± 0.02	0.70 ± 0.05	***
Epithelium	0.84 ± 0.03	0.60 ± 0.04	***		0.97 ± 0.01	0.87 ± 0.02	***
Muscularis mucosae	0.90 ± 0.02	0.81 ± 0.04	*		0.99 ± 0.00	0.95 ± 0.01	*
Muscularis externa	0.92 ± 0.01	0.89 ± 0.03	NS		0.99 ± 0.00	0.98 ± 0.01	NS

^1^Indices were calculated using the Image J (http://rsbweb.nih.gov/ij/) image analysis software.

NS > 0.05; *P < 0.05; **P < 0.01; ***P < 0.001.

#### Area Analysis (Table 2).

The total area of the cross-section of the gastric isthmus (including the lumen) was significantly smaller in D− than in D+. The lumen area was 4 times smaller in D− than in D+ (respectively 0.06 vs. 0.24 cm^2^, n = 10, *P* < 0.001), and tissue area of D− accounted for 72% of that of D+ (respectively 0.51 vs. 0.69 cm^2^, n = 10, *P* < 0.01). Absolute areas of epithelium and muscularis externa layers were also significantly smaller in D− than in the D+ isthmus. At the opposite, mucous and muscularis mucosae absolute areas did not differ significantly between lines (*P* > 0.05). The absolute area of the mucosa layer (mucous + epithelium + muscularis mucosae) was significantly smaller in D− than in D+ (*P* < 0.05, D− to D+ ratio = 0.74, not shown).

**Figure 3.**
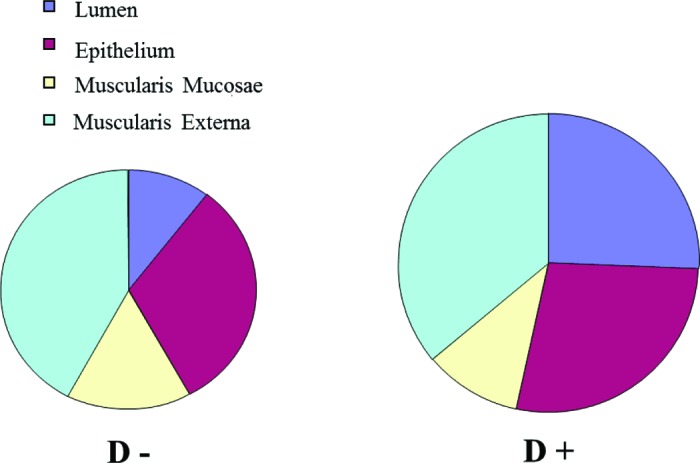
Mean relative area (ratio of the area of gastric isthmus cross-section to the total isthmus cross-section area from D+ and D− (more feed efficient and less feed efficient, respectively) line chickens. Area was calculated using the Image J (http://rsbweb.nih.gov/ij/) image analysis software. Color version available in the online PDF.

The relative area (ratio to total surface of the cross-section) is presented in Table 2 and in Figure 3. The lumen occupied 11% of the whole surface of the section in D− against 24% in D+ (*P* < 0.01). The relative proportion of the mucosa layer was higher in D− than in D+ (respectively 47 and 39% of the total surface of the section, *P* < 0.01). The muscularis mucosae area was relatively more developed in D− than in D+, occupying, respectively, 16 and 11% of the whole surface of the section (*P* < 0.01). The relative area of the epithelium and muscularis externa did not differ between lines. The muscularis externa layer occupied similar relative area in D− and D+, averaging 39% of the whole surface of the section (i.e., 42% in D− and 37% in D+, *P* > 0.05). Similar results were obtained when values were reported to whole tissue area excluding the lumen area (not shown).

#### Morphological Analysis (Table 3).

The circularity index varied from 0.41 (lumen) to 0.89 (muscularis externa) for D− versus 0.67 (lumen) to 0.92 (muscularis externa) for D+ (Table 3). External contours of muscularis externa of the section trended toward a circle in D− as in D+ chickens (*P* > 0.05). The ratios of D− to D+ circularity indicators were 0.56, 0.89, and 0.96, respectively, for contours of the mucous, muscularis mucosae, and muscularis externa layers. The solidity index evolved from 0.65 (lumen) to 0.98 (muscularis externa) for D− versus 0.90 to 0.99 for D+ chickens (Table 3). This index accounts for a more or less folded profile. The ratios of D− to D+ solidity index were 0.76, 0.96, and 1.00, respectively, for contours of the mucous, muscularis mucosae, and muscularis externa layers.

## DISCUSSION

Previous results on 2 chicken lines (D+/D−) selected on AME_n_ converged to the conclusion that the gastric compartment plays a major role in inducing different digestive capacities between lines (Garcia et al., 2007; Rougière et al., 2009; de Verdal et al., 2010, 2011; Rougière and Carré, 2010; Rougière et al., 2012). To go further, we characterized the gastric isthmus located between the proventriculus and the gizzard, a region expected to regulate gastric motility in birds (Chaplin and Duke, 1988; Lecoin et al., 1996; Hall and Duke, 2000), which had been shown to differ between D+ and D− lines (Rougière and Carré, 2010). We confirmed that selection of chicks modified the morphology of the upper part of the digestive tract, namely the gizzard and the proventriculus being smaller and lighter in D− than in D+ chicks. We further report histological differences at the gastric isthmus level through cross-section histological analysis. The whole area of the isthmus cross-section was consistently significantly smaller in D− than in D+. The main difference concerned the lumen, the surface of which was 4 times smaller in D− than in D+. The lumen occupied 11% of the whole D− isthmus surface against 24% for D+. The lumen side of the isthmus mucosa was significantly more oval and more folded in D− than in D+ chickens and the mucosa relative area was higher in D− than in D+. The muscular part of the mucosa (muscularis mucosae) was significantly more developed in D− than in D+ chickens.

In a recent study, de Verdal et al. (2010) reported changes in the morphology and histology of the small intestine in the D− line compared with the D+ line. They showed that the muscular layer (tunica muscularis) of all intestinal segments (duodenum, jejunum, and ileum) was significantly thicker (+17 to +24% in D− compared with D+) and the number of goblet cells per villus was significantly higher for D− birds than in D+ birds in the jejunum and ileum (respectively +27 to +34%). The authors suggested that intestinal adaptation revealed by visceral organ weight and length and histological modifications in D− birds could be viewed as an attempt to compensate for the low functionality of the gastric compartment. Modifications of the gastric isthmus mucosa profile and development of the gastric isthmus muscularis mucosae reported in the present study suggest that some similar regulatory mechanism(s) are responsible for morphological modifications of the gastric and intestinal compartments in D− line and D+ line. Increase in the muscular layer area (muscularis mucosae) and folding of the mucosa in D− chicks are consistent with increased gizzard contractile activity reported in D− compared with D+ chickens (Rougière et al., 2012). The present results confirm, illustrate, and give new insight on the central role of the gizzard in differences between D+ and D− lines (Péron et al., 2006; Garcia et al., 2007; Rougière et al., 2009; Rougière and Carré, 2010; de Verdal et al., 2011; Rougière et al., 2012). It focuses on the gastric isthmus, which is expected to play a key role in gastric motor regulation, as suggested by the presence of c-kit-positive cells at the junction between proventriculus and gizzard in the avian bowel (Lecoin et al., 1996). Further study on mechanism regulating motor activity and trophicity of the gastric isthmus in the divergent lines selected for digestive efficiency is clearly warranted.
